# Novel Genes Affecting the Interaction between the Cabbage Whitefly and *Arabidopsis* Uncovered by Genome-Wide Association Mapping

**DOI:** 10.1371/journal.pone.0145124

**Published:** 2015-12-23

**Authors:** Colette Broekgaarden, Johan Bucher, Johanna Bac-Molenaar, Joost J. B. Keurentjes, Willem Kruijer, Roeland E. Voorrips, Ben Vosman

**Affiliations:** 1 Wageningen UR Plant Breeding, Wageningen University, Wageningen, The Netherlands; 2 Plant-Microbe Interactions, Department of Biology, Utrecht University, Utrecht, The Netherlands; 3 Laboratory of Plant Physiology, Wageningen University, Wageningen, The Netherlands; 4 Laboratory of Genetics, Wageningen University, Wageningen, Wageningen, The Netherlands; 5 Biometris–Applied Statistics, Department of Plant Science, Wageningen University, Wageningen, The Netherlands; National Institute of Plant Genome Research (NIPGR), INDIA

## Abstract

Plants have evolved a variety of ways to defend themselves against biotic attackers. This has resulted in the presence of substantial variation in defense mechanisms among plants, even within a species. Genome-wide association (GWA) mapping is a useful tool to study the genetic architecture of traits, but has so far only had limited exploitation in studies of plant defense. Here, we study the genetic architecture of defense against the phloem-feeding insect cabbage whitefly (*Aleyrodes proletella*) in *Arabidopsis thaliana*. We determined whitefly performance, i.e. the survival and reproduction of whitefly females, on 360 worldwide selected natural accessions and subsequently performed GWA mapping using 214,051 SNPs. Substantial variation for whitefly adult survival and oviposition rate (number of eggs laid per female per day) was observed between the accessions. We identified 39 candidate SNPs for either whitefly adult survival or oviposition rate, all with relatively small effects, underpinning the complex architecture of defense traits. Among the corresponding candidate genes, i.e. genes in linkage disequilibrium (LD) with candidate SNPs, none have previously been identified as a gene playing a role in the interaction between plants and phloem-feeding insects. Whitefly performance on knock-out mutants of a number of candidate genes was significantly affected, validating the potential of GWA mapping for novel gene discovery in plant-insect interactions. Our results show that GWA analysis is a very useful tool to gain insight into the genetic architecture of plant defense against herbivorous insects, i.e. we identified and validated several genes affecting whitefly performance that have not previously been related to plant defense against herbivorous insects.

## Introduction

In nature, plants are constantly exposed to a wide range of herbivorous insects that can severely affect their growth and reproduction. Adaptation of plants, driven by differences in herbivore selection pressure, has resulted in natural variation for defense mechanisms [1,2]. This variation is thought to be maintained by trade-offs between the benefits of reducing herbivore damage and the costs of defense [3]. Plant traits that serve as an effective defense against herbivores have been shown to evolve rapidly when herbivores are present, whereas such traits are lost from the population in the absence of herbivores [4,5]. Certain traits that plants have evolved to counteract herbivore attack are involved in direct defense, i.e. physical and/or chemical barriers that negatively affect the performance of the attacker [6]. Physical barriers, such as trichomes, hinder or prevent insects from moving or feeding on a plant whereas toxic or anti-feedant compounds form a chemical barrier to herbivores. These traits can alter the physiology of herbivorous insects resulting in reduced growth rate, adult size, survival probability and reproduction success [7].

In several plant species variation for direct defense traits has been found [8,9,10,11,12]. This variation has been used to develop segregating biparental populations to enable the identification of quantitative trait loci (QTLs) [13]. Several QTLs controlling defense against insects have been mapped, cloned and characterized [14,15,16]. For example, *CYP81F2* has been identified as the gene underlying a metabolic QTL in *Arabidopsis thaliana* that contributes to defense against aphids [17]. At present, genome wide association (GWA) mapping has become a popular approach to study the genetic architecture, i.e. statistically link variation in a specific trait to polymorphic molecular markers, of both qualitative and quantitative plant traits. In contrast to traditional QTL mapping in bi-parental populations, GWA mapping is a population based method and, as a wide diversity of material is used, it is expected to uncover more genes and allelic diversity contributing to (polygenic) traits [18,19]. GWA mapping has been used successfully to identify loci controlling several traits involved in plant-attacker interactions, such as defense against pathogens [20] and defensive secondary metabolites [21]. Recently, Samayoa *et al*. (2015) were able to identify genomic regions in maize that play a role in defense against the corn borer, a leaf chewing insect [22]. However, studies that directly associate genomic regions with the performance of other insect types or species are lacking. Moreover, validation and characterization of candidate genes explaining observed variation for defense against insects is lacking.

Because *A*. *thaliana* has a worldwide distribution and therefore encounters diverse ecological conditions, it is a very appropriate species to study natural variation for adaptive traits [23]. So far, natural variation among *A*. *thaliana* accessions has been found for insect behavior and performance [24,25] as well as for defensive secondary metabolites [12]. Using traditional linkage mapping or reverse genetics with knock-out or overexpression mutants, several genes have been identified that affect *A*. *thaliana* defense towards insects [14,15,16,26,27,28]. For example, *MAM1* has been shown to cause variation in the side-chain length of aliphatic glucosinolates, which are defensive secondary metabolites in brassicaceous plants [29]. Additionally, *PAD4* is a well-known example of a gene that promotes *A*. *thaliana* defense towards aphids [30]. In this study we focused on the defense of *A*. *thaliana* against the cabbage whitefly *Aleyrodes proletella*. To date, little is known about the interaction between *A*. *thaliana* and this phloem-feeding insect. *Aleyrodes proletella* is a pest in *Brassica oleracea* crops and difficult to control due to its short generation time and high reproduction rate. Most nymphal stages are immobile and continuously feed at the same location during their development [31]. The objectives of this study were to investigate (1) whether there is variation for defense against the cabbage whitefly *A*. *proletella* among a large set of *A*. *thaliana* accessions, (2) to obtain information on the genomic regions underlying this variation, and (3) to get insight in the effects individual genes may have on whitefly performance.

## Results

### Natural variation in whitefly performance

**Fig 1:**
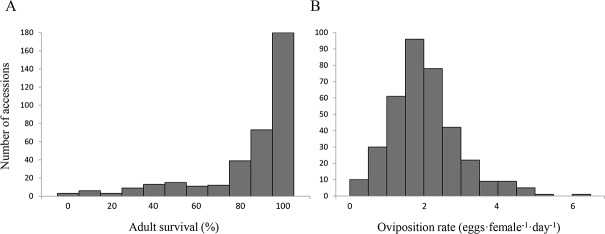
Frequency of *Arabidopsis thaliana* accessions against the performance of *Aleyrodes proletella*. Shown is the number of accessions with a certain adult survival (A) or oviposition rate (B) based on the averages of four replicate experiments. Each replicate consisted of three incomplete blocks of 120 accessions and additionally five reference accessions (see Materials & Methods).

To detect variation for direct defense against *A*. *proletella*, whitefly adult survival and oviposition rate were monitored using a no-choice assay on 360 accessions of *A*. *thaliana* grown under controlled conditions. Four independent experiments were done, each containing the complete collection of 360 *A*. *thaliana* accessions. Each replicate consisted of three incomplete blocks of 120 accessions and additionally five reference accessions (see Materials & Methods). The broad-sense heritability was estimated at 0.71 for adult survival and 0.74 for oviposition rate. Variation for adult survival was relatively small as most whiteflies survived on the majority of accessions (Fig 1; S1 Table). For oviposition rate, substantial variation was observed, ranging from zero to 6.5 eggs·female^-1^·day^-1^ (Fig 1; S1 Table). The two whitefly performance parameters were positively correlated with each other (*r* = 0.64, *P* < 0.001) but not with latitude or longitude of the origin of collection of the accessions (-0.045 < *r* < 0.04, *P* > 0.4).

### GWA mapping

**Fig 2:**
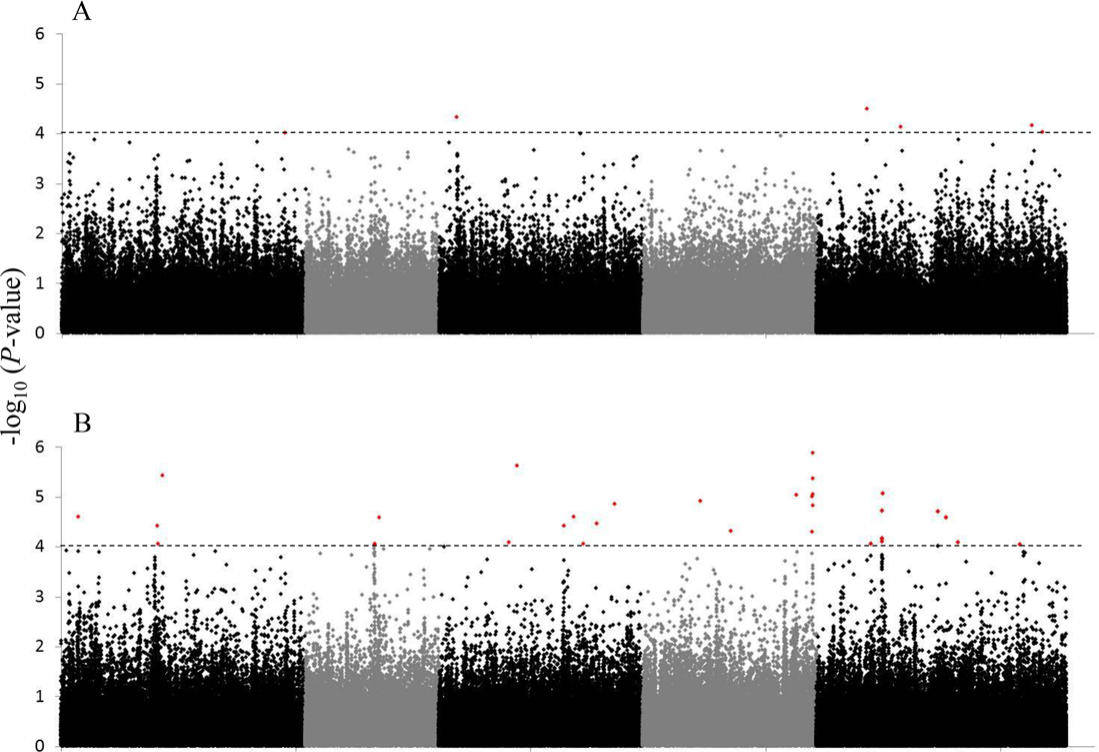
Manhattan plots for GWA of variation in *Aleyrodes proletella* performance. Associations between SNPs and adult survival (A) or oviposition rate (B) are shown. Each SNP is represented by a single dot with the candidate SNPs (-log_10_(*P*) > 4, MAF > 5%; horizontal dashed line) plotted in red. SNPs with a MAF < 5% were not included in the analysis. *Arabidopsis thaliana* chromosomes 1–5 are shown in contrasting colors from left to right on the x-axis.

Using a set of 214,051 SNPs available for this collection of 360 accessions [20,32], GWA mapping was carried out using a model that corrects for genetic relatedness (EMMAx). SNPs with a minor allele frequency (MAF) < 5% were not considered in the model because of possibly elevated false-discovery rates [20]. Six and 33 SNPs with *P* < 10^−4^ and MAF > 5% were considered as candidate SNPs for adult survival and oviposition rate, respectively, explaining 4.4% to 6.7% of the phenotypic variation (Fig 2, Table 1). There was no overlap in associated SNPs between whitefly adult survival and oviposition rate.

**Table 1:** SNPs significantly associated with *A*. *proletella* adult survival or oviposition rate.

Chromosome	Position (bp)	Locus^1^	-log_10_(*P* value)	Explained variance (%)
Survival				
1	27445448	S1	4.01	4.4
3	2173384	S2	4.31	4.8
5	5982121	S3	4.49	5.0
5	9120976	S4	4.12	4.5
5	22804782	S5	4.15	4.6
5	24055182	S6	4.01	4.4
Oviposition rate				
1	2253521	OR1	4.58	5.1
1	11589257	OR2	4.41	4.9
1	11687888	OR3	4.05	4.4
1	12109439	OR4	5.42	6.1
2	9820793	OR5	4.04	4.4
2	9838911	OR6	4.02	4.4
2	10544649	OR7	4.57	5.1
3	8316417	OR8	4.07	4.5
3	9169015	OR9	5.62	6.4
3	14680659	OR10	4.40	4.9
3	15602919	OR11	4.59	5.1
3	16587682	OR12	4.05	4.4
3	17895650	OR13	4.45	4.9
3	19981555	OR14	4.85	5.4
4	6432812	OR15	4.90	5.5
4	8757099	OR16	4.30	4.7
4	15998778	OR17	5.03	5.6
4	18054888	OR18	4.28	4.7
4	18058798	OR18	5.00	5.6
4	18106794	OR19	5.35	6.0
4	18115709	OR19	4.81	5.4
4	18149448	OR20	5.87	6.7
4	18158297	OR20	5.04	5.6
5	6424075	OR21	4.05	4.4
5	7469286	OR22	4.16	4.6
5	7469355	OR22	4.16	4.6
5	7470772	OR22	4.71	5.2
5	7472032	OR22	4.09	4.5
5	7479469	OR22	5.06	5.7
5	13926089	OR23	4.69	5.2
5	14894815	OR24	4.58	5.1
5	15897271	OR25	4.07	4.5
5	21435322	OR26	4.03	4.4

SNPs with *P* < 10^−4^, MAF > 5% are shown.

^1^S, survival; OR, oviposition rate. SNPs located in sufficient LD (Pearson r^2^ > 0.5) were assigned to the same locus.

Genes located at or within 10 kb (because LD in *A*. *thaliana* extends over 5 to 10 kb [32]) and in sufficient LD (Pearson r^2^ > 0.5) with at least one candidate SNP were considered candidate genes causal for the observed SNP effects. Annotations of the 57 candidate genes were derived from the Gene Ontology tool at The *Arabidopsis* Information Resource (TAIR; [33] (S2 Table). Interestingly, none of the 57 candidate genes have previously been associated with plant-whitefly interactions. Many genes of unknown function were present among the candidate genes, i.e. 50% and 32% for respectively adult survival and oviposition rate. Candidate genes for which a predicted function is available are involved in many different processes. Several of these genes are potentially involved in processes that have been related to defense against phloem-feeding insects, such as cell wall modification, secondary metabolism and hormone signaling [34,35] (S2 Table). One of the significant SNPs on chromosome 3, i.e. locus OR9, is located in *CYP82G1* that has been shown to play a role in *A*. *thaliana* defense against caterpillars [36].

### Validation of individual genes associated with whitefly performance

**Fig 3:**
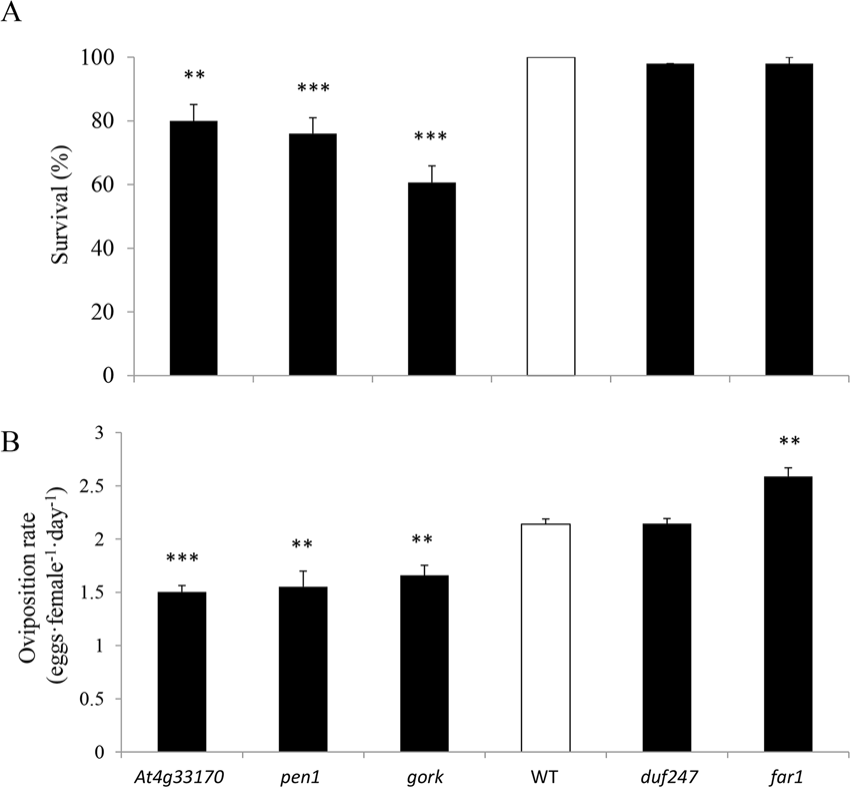
*Aleyrodes proletella* performance on mutant and wild type plants. Seven days after infestation, adult survival (A; in percentages) and oviposition rate (B; expressed as the number of eggs laid per female per day) were monitored on wild type Col-0 (WT; white bar) and T-DNA mutant lines (black bars). Ten plants per genotype were used. The experiment was repeated independently with similar results. Stars indicate significant differences compared with the wild type (Mann-Whitney U test; * *P* < 0.05, ** *P* < 0.01, *** *P* < 0.001).

To determine whether genes identified from the GWA analysis actually influence whitefly performance, we tested the knock-out effect of five candidate genes for oviposition rate using T-DNA insertion mutants (S2 Table). These mutants were randomly selected based on their predicted function and the availability of mutant lines from the Arabidopsis stock center [37]. Whitefly performance was determined on the mutants and compared to that on wild type Col-0. A T-DNA insertion in *PEN1* (*At4g15340*), a gene involved in biosynthesis of the secondary metabolite triterpenoid [38], resulted in a significant reduction of adult survival as well as oviposition rate (Fig 3). Similar results were obtained on mutant plants having a T-DNA insertion in *GORK* (*At5g37500*), a gene involved in transpiration [39] (Fig 3). Functional knock down of *At4g33170*, a gene of unknown function, also resulted in reduced adult survival and oviposition rate (Fig 3). Whitefly performance on a knockout mutant of *At5g22540*, a protein of unknown function containing a *duf247* domain probably involved in ethylene biosynthesis, was similar to that on the wild type. Knocking out *FAR1* (*At5g22500*), which is in LD with *At5g22540*, did show a significant effect on oviposition rate (Fig 3). Female whiteflies laid significantly more eggs on mutants having a T-DNA insertion in *FAR1*, a gene involved in the generation of fatty alcohols [40], than on wild type plants. Adult survival was not affected on this mutant (Fig 3).

## Discussion

### Variation in whitefly performance is predominantly based on minor effect genes

*Arabidopsis thaliana* has a world-wide distribution and faces many biotic and abiotic challenges during its life cycle. We show, for the first time, variation for performance of the cabbage whitefly, *A*. *proletella*, on a large set of *A*. *thaliana* accessions originating from different geographical locations. The high heritability found for both whitefly adult survival and oviposition rate (0.71 and 0.74 respectively) indicates that the observed variation is mainly caused by genetic factors. It also reflects the reliability and reproducibility of our experiments.

GWA mapping resulted in the identification of six and 33 candidate SNPs for whitefly adult survival and oviposition rate, respectively. The relatively low number of SNPs associated with adult survival may be due to the skewed distribution towards susceptibility, i.e. on most of the accessions all adult whiteflies could survive for seven days. Despite the correlation between adult survival and oviposition rate, there was no overlap in associated SNPs between the two parameters. These data suggest that certain traits can affect oviposition rate without influencing adult survival, a phenomenon that has previously been shown for the silverleaf whitefly *Bemisia tabaci* on wild tomato plants [41]. It is conceivable that certain plant traits negatively affect the reproduction of whitefly adults but are not lethal to them. For example, high levels of glucosinolates have been shown to negatively affect whitefly oviposition rate but did not affect adult survival on *A*. *thaliana* [42].

*Arabidopsis thaliana* genes involved in plant-insect interactions have mainly been identified in a few common laboratory accessions [24,26,43]. This limits the possibility to study genetic variation and the identification of genes that are absent or not functional in these laboratory accessions. Using a large collection of accessions, we identified 39 SNPs associated with whitefly performance. In spite of the high heritability of the two traits, each individual SNP only explains between 4.4% and 7.6% of the observed phenotypic variation, suggesting that the genetic architecture of defense is complex, with many contributing loci. Such quantitative traits for defense against insects are quite commonly found in different plant species [8] and difficult to dissect genetically [44]. Additionally, it is possible that low frequency functional alleles that have little influence on the population as a whole are present in some accessions [44]. Such genes or alleles are not detected in our GWA analysis as we used a 5% minor allele frequency cut-off. This also implies that although we did not identify genes with a major effect on whitefly performance in our GWA analysis, such genes may be present in *A*. *thaliana*. This is supported by the observation that adult survival and/or oviposition rate was strongly reduced on a small number of accessions. Further studies using populations derived from bi-parental crosses with these accessions may be used to identify low frequency functional alleles.

### Novel genes affecting whitefly performance

Using GWA mapping, we have identified several candidate SNPs for adult survival and oviposition rate of *A*. *proletella*. Interestingly, only one of the corresponding candidate genes for oviposition rate, *CYP82G1*, has previously been related to defense against insects. *CYP82G1* is known to be involved in the production of the caterpillar-induced homoterpene volatile TMTT [36] and volatile blends containing TMTT have been shown to attract the parasitic wasp *Cotesia rubecula* that parasitizes *Pieris rapae* larvae [45]. Well known genes that have been shown to affect the performance of phloem-feeding aphids or whiteflies [26,46] were not detected in our GWA analysis. Some of the previously identified genes are JA or SA regulatory genes involved in the biosynthesis of these hormones, e.g. *CEV1* [47], or playing a role in unlocking the down-stream responses, e.g. *NPR1* [48]. Possibly, there is very little variation for these genes among the accessions and therefore they were not identified using GWA mapping. Additionally, some previously identified defense genes have been shown to affect aphid performance but have no influence on the behavior of *B*. *tabaci* whiteflies, such as *COI1* and *PAD4* [46,49], and may similarly also not affect the performance of *A*. *proletella*. Finally, it should be noted that some of the previously identified genes may affect other *A*. *proletella* performance parameters that we did not take into account in our study.

One way to validate the identified candidate genes is to use knockout mutants. As T-DNA insertion lines in the Col-0 background are readily available for many genes [37], they are a useful resource for preliminary identification/validation. Because oviposition rate on Col-0 was average compared with all the other accessions (1.8 eggs·female^-1^·day^-1^), using such lines may quickly provide valuable information on the possible effect that individual candidate genes may have on whitefly performance. Five mutants having a T-DNA in a candidate gene for oviposition rate were tested and four of them significantly affected whitefly performance. Oviposition rate and, interestingly, adult survival was reduced on these mutants while these genes were not identified in the GWA analysis for the latter parameter. Additionally, the effect of the T-DNA insertion was much larger than expected based on the explained variation of the corresponding candidate SNPs. This suggests that complete silencing of a gene has a much bigger effect on whitefly performance than a single or a few point mutations. The explained variance of a candidate SNP identified using GWA mapping is probably low due to the fact that there are many loci with an effect on whitefly performance, all contributing to the variance to some extent. Conversely, in the mutant lines there are no interfering effects of genes other than the one containing the T-DNA, meaning that all the variation is due to this one locus and the residual variance.

The reduced oviposition rate on these four mutants indicates that the functional copy of the gene in question acts as a repressor of defenses or contributes to susceptibility. Such genes have been identified before in *A*. *thaliana* affecting aphids [43,49] and whiteflies [46]. Among the tested genes was *PEN1*, a gene previously shown to promote fungal penetration resistance, i.e. silencing this gene in *A*. *thaliana* leads to increased susceptibility to certain fungi [50,51]. However, we found the opposite effect for whitefly performance on a mutant containing a T-DNA in *PEN1*, suggesting a trade-off between resistance to different attackers. The mutant containing a T-DNA insertion in *FAR1* positively affected oviposition rate and had no effect on adult survival. The increased susceptibility on this mutant indicates that *FAR1* contributes to plant defense. This gene is induced upon pathogen attack or mechanical wounding thereby activating suberization of primary cell walls, a process that likely serves to seal off the tissue to prevent further damage [40], and may thus be related to defense against herbivorous insects. The exact role of *PEN1* and *FAR1* as well as *GORK* and *At4g33170* in the plant-whitefly interaction remains to be established. Additionally, it will be interesting to investigate whether the orthologues of these genes in a crop plant, e.g. cabbage, also affect whitefly performance and whether or not this is accompanied by a yield penalty.

### Evolution of genetic variation for whitefly performance

Apart from identifying genes involved in the plant-whitefly interaction, studying genetic variation may also help to understand how resistance has evolved, e.g. if it is shaped by co-evolutionary processes or that the observed variation is a side effect of adaptation to other environmental factors [52]. A hallmark of co-evolution can be a geographic cline of certain genotypes that follow the distribution pattern of pests or pathogens. In this case one would expect that the occurrence of resistance traits is geographically structured, e.g. resistance is found in areas where whitefly and plant have occurred together for a long time [53]. In our study, there was no correlation between whitefly performance and the geographic coordinates latitude or longitude of the origin of collection of the accessions, suggesting that co-evolution between *A*. *thaliana* and whitefly did not occur on a global scale. However, it should be noted that insects can have very patchy distributions and studying geographic location on a large scale may overlook co-evolution that occurred in smaller patches of habitat [54]. Additionally, all accessions were grown under the same environmental conditions while they originate from different areas with different environmental conditions. It is therefore possible that certain accessions do not perform optimally under our experimental conditions, which may have consequences for the expression of defenses and obscure correlations with geographic coordinates. In nature, plants are exposed to a wide range of biotic and abiotic factors that can all modify defense characteristics [55]. For example, environmental stresses such as temperature fluctuations, nutrient deficiency or other abiotic factors may result in selections that also affect pathogen/herbivorous insect interactions, e.g. via different patterns of resource allocation in plants causing spatial variation in defense traits [56]. This is supported by the observation that knocking-out *GORK*, a gene involved in transpiration, has an effect on whitefly performance. Further studies are needed to investigate whether the observed natural variation is due to co-evolution between *A*. *thaliana* and *A*. *proletella* or whether it is a side effect of adaptation to other environmental factors.

### Conclusion

GWA mapping has been shown to be powerful for dissecting the genetic network of quantitative traits as well as identifying novel genes related to those traits [20]. In this study, we show that GWA mapping is an excellent tool to gain insight into the genetic architecture of plant defense against herbivorous insects. Thirty-eight candidate SNPs for natural variation in defense against the cabbage whitefly were identified that had so far not been related to the interaction between plant and phloem-feeding insects. Functional validation showed that four candidate genes affect whitefly performance indicating the reliability of our study.

## Materials and Methods

### Plant material and cultivation

For this study we used a set of 360 *Arabidopsis thaliana* accessions [57,58]. Stock numbers and detailed information for accessions are listed in S1 Table. Seeds of T-DNA insertion mutants were obtained from the European Arabidopsis Stock Center (NASC; http://arabidopsis.info/; [37]. Mutant plants were checked for the homozygous presence of the T-DNA insertion by performing a PCR reaction on genomic DNA using a left border primer of the T-DNA insertion (LBb1.3 5’-ATT TTG CCG ATT TCG GAA C-3’) and gene specific primers (S3 Table).

Cultivation and experiments were conducted in a climate chamber (20 ± 1°C) with 10 hours of light (200 μE.m^-2^.sec^-1^) at a relative humidity of 65%. Before germination, seeds were stratified on wet filter paper for three days at 10°C in the dark. For GWA mapping experiments, seeds were sown on 4x4 cm Rockwool plugs (MM40/40, Grodan B.V.) and watered daily with 1 g/l Hyponex fertilizer (NPK = 7:6:19) using a flooding system. For experiments with T-DNA mutants, seeds were sown in 60 ml pots containing soil sterilized by gamma irradiation containing vermiculite (Horticoop®). Plants were watered every other day. No chemical control for pests or diseases was applied.

### Insect rearing

Cabbage whiteflies, *Aleyrodes proletella*, were reared on Brussels sprouts (*B*. *oleracea* var. *gemmifera* cv. Cyrus). The whitefly population originated from adults collected in 2008 from a white cabbage field in Wageningen, the Netherlands (N 51° 57’, E 5° 38’) and the rearing was situated in a climate chamber at 20±2°C with an L16:D8 photoperiod and 40–60% RH [59]. Insects were reared under conditions in which there was always sufficient foliage for feeding and oviposition.

### Whitefly performance

Five week old plants were used to examine whitefly performance. One clip cage (Ø 2 cm, height 2x1.2 cm) containing five females was placed on a young leaf of each plant. Whiteflies were briefly (< 30 min) anaesthetized with carbon dioxide (80% N_2_:10% H_2_:10% CO_2_; Linde Gas Benelux) to enable selection and transfer of females. Seven days after infestation, the number of living and dead females as well as the number of eggs was counted. Subsequently, adult survival and oviposition rate (eggs·female^-1^·day^-1^) were calculated per plant.

### Experimental design

In the GWA mapping experiments, four independent replicates were performed, each containing a single plant of every accession. Each replicate consisted of three incomplete blocks of 120 accessions. Five accessions (Col-0, Ler-1, WS-0, Cvi-0 and Kin-0) were additionally present in each block to serve as controls, but the data obtained for these plants were not used in further analyses. Prior to estimation of heritability and GWA mapping, the individual plant observations were transformed, using the arcsine-square root-transformation for adult survival and a log10 (*x* + 1) transformation for oviposition rate. Pearson correlation tests were used to determine the relatedness between whitefly performance parameters as well as between whitefly performance parameters and longitude/latitude.

T-DNA mutants were tested with their corresponding wild type Columbia-0 in a randomized complete block design with 10 plants per genotype. Mann-Whitney U tests was used to test the significance of differences between whitefly performance on mutant and wild type plants. The experiment was repeated once to obtain data from two independent experiments.

### Heritability

We used the R-package ‘heritability’ [60] to estimate broad-sense heritability. The estimate is given by

$$H^{2}=\frac{Vg}{Vg+Ve},\;with\;Vg\;=\;\frac{MS(accession)-MS(error)}{r}and\;Ve\;=\;MS(error),$$

where Vg and Ve represent the genetic and residual variance, and r is the average number of replicates (slightly less than 4, due to missing values). The terms MS(accession) and MS(error) are the mean sums of squares for respectively accession and error, obtained from an analysis of variance, including (fixed) effects for replicate and accession.

### Genome-wide association mapping

The *A*. *thaliana* accessions have previously been genotyped using a 250K SNP chip [20] and this data was used for GWA mapping using the software scan_GLS [60]. For both traits, we first analyzed the phenotypic data from all replicated by fitting the mixed model

Yij=Mean+Accession(i)+Replicate(j)+Block(withinreplicatej)+error(ij),

with fixed effects for accession (i = 1,…,360) and replicate (j = 1,2,3,4) and a random effect for incomplete blocks within replicates. GWA mapping was performed on the accession means, i.e. the best linear unbiased estimator (BLUE) for Accession in the mixed model above. For these we assumed the mixed model

Yi=μ+xiβ+Gi+Ei,

where μ is the population mean, x_i_ is the SNP-score of accession i (zero or one), β is the SNP effect, and G_i_ and E_i_ are random effects. The vector of genetic effects (G_1_,…,G_360_) has a multivariate normal distribution with zero mean and covariance σ_A_^2^ K, where K is a genetic relatedness matrix derived from all SNPs and σ_A_^2^ > 0 is the additive genetic variance. The residual effects E_i_ are assumed to have independent normal distributions, with variance σ_E_^2^. As in Kang [61], we first estimated the parameters σ_A_^2^ and σ_E_^2^ using a model without a SNP (i.e. Y_i_ = μ + G_i_ + E_i_). Next, to estimate the SNP-effect β, we fitted model (1) for each SNP in turn, given the estimates for σ_A_^2^ and σ_E_^2^ obtained in the first step. The relatedness matrix K was defined as the identity by state matrix derived from all SNP markers.

From the GWAS analysis, SNPs with *P* < 10^−4^ and MAF > 5% were considered as candidate SNPs for adult survival and oviposition rate. Next, the set of candidate SNPs was extended with SNPs located within 10 kb (based on the observation that LD in *A*. *thaliana* decays over 5–10 kb) and in sufficient LD (Pearson r^2^ > 0.5) with at least one of the candidate SNPs. Genes located at or near the candidate SNPs were considered as candidate genes playing a role in the interaction between the cabbage whitefly and *A*. *thaliana*.

## Supporting Information

S1 Table Whitefly performance on 360 *Arabidopsis thaliana* accessions originating from different geographic locations. (XLSX)

S2 Table List of genes in LD with SNPs associated with adult survival or oviposition rate. (XLSX)

S3 Table List of gene specific primers used to confirm homozygosity of T-DNA insertion mutants. (XLSX)
